# Chick Embryo: A Preclinical Model for Understanding Ischemia-Reperfusion Mechanism

**DOI:** 10.3389/fphar.2018.01034

**Published:** 2018-09-21

**Authors:** Eram Fauzia, Tarun Kumar Barbhuyan, Amit Kumar Shrivastava, Manish Kumar, Paarth Garg, Mohsin Ali Khan, Avril A. B. Robertson, Syed Shadab Raza

**Affiliations:** ^1^Laboratory for Stem Cell and Restorative Neurology, Department of Biotechnology, Era’s Lucknow Medical College and Hospital, Era University, Lucknow, India; ^2^Era’s Lucknow Medical College and Hospital, Era University, Lucknow, India; ^3^School of Chemistry and Molecular Biosciences, The University of Queensland, Brisbane, QLD, Australia; ^4^Department of Stem Cell Biology and Regenerative Medicine, Era University, Lucknow, India

**Keywords:** ischemia-reperfusion, chick embryo, Hook I/R model, Doppler blood flow imaging, autophagy, NLRP3 inflammasome, MCC950

## Abstract

Ischemia-reperfusion (I/R)-related disorders, such as stroke, myocardial infarction, and peripheral vascular disease, are among the most frequent causes of disease and death. Tissue injury or death may result from the initial ischemic insult, primarily determined by the magnitude and duration of the interruption in blood supply and then by the subsequent reperfusion-induced damage. Various *in vitro* and *in vivo* models are currently available to study I/R mechanism in the brain and other tissues. However, thus far, no *in ovo* I/R model has been reported for understanding the I/R mechanisms and for faster drug screening. Here, we developed an *in ovo* Hook model of I/R by occluding and releasing the right vitelline artery of a chick embryo at 72 h of development. To validate the model and elucidate various underlying survival and death mechanisms, we employed imaging (Doppler blood flow imaging), biochemical, and blotting techniques and evaluated the cell death mechanism: autophagy and inflammation caused by I/R. In conclusion, the present model is useful in parallel with established *in vitro* and *in vivo* I/R models to understand the mechanisms of I/R development and its treatment.

## Introduction

The incidence of ischemia-reperfusion (I/R) injury is high, and its pathogenesis involves complex, multifactorial, and interrelated processes. I/R contributes to the pathophysiology of stroke, myocardial infarction, peripheral vascular insufficiency, and other thrombotic events. Prolonged ischemia results in detrimental cellular metabolic and ultrastructural changes. Thus, to minimize or prevent irreversible cellular injury, restoring blood supply is essential. Notably, reperfusion can augment the tissue injury compared with that produced by ischemia alone (Mathes et al., 2016; Tejada et al., 2016; Silachev et al., 2017). Thus, prompt revascularization and blood flow restoration, with minimal damage to the reperfused area, remain the mainstay of all current therapeutic approaches for I/R (Linfante and Cipolla, 2016; Strand-Amundsen et al., 2018; Xiong et al., 2018; Yan et al., 2018). To mimic the aforementioned mechanism, suitable models closely resembling human pathology in clinical conditions are needed, that can contribute to our understanding of the mechanisms underlying I/R injury (Milcan et al., 2004; Lai et al., 2006; Kalogeris et al., 2012, 2016; Horvath et al., 2016; Ross et al., 2016; McBride and Zhang, 2017). Such models aid the understanding of I/R mechanisms and are also used in drug testing pipelines; ultimately translating to improved patient care.

In the last three decades, several critical factors that can act in concert to mediate the detrimental effect of I/R injury have been identified. However, till date no treatment directed to I/R injury has shown to lead to an improvement in clinical outcomes. This is primarily because of the lack of our complete understanding of the complexity of disease progression, and secondarily because of inappropriate research model selection. Currently, multiple species, including non-human primates, rodents, felines, and certain avian species, are used in I/R research. The disparity between the results obtained using these models and the results of clinical trials, in humans, have led to the development of newer experimental model (Allen et al., 2005; Schmeer et al., 2008; Anvret et al., 2012; Xu et al., 2014; Gonzalez et al., 2015; Huang et al., 2016; Sommer, 2017; Yang et al., 2018). In this study, we used chick embryos as an alternative model to study the underlying mechanism of I/R injury.

**FIGURE 1:**
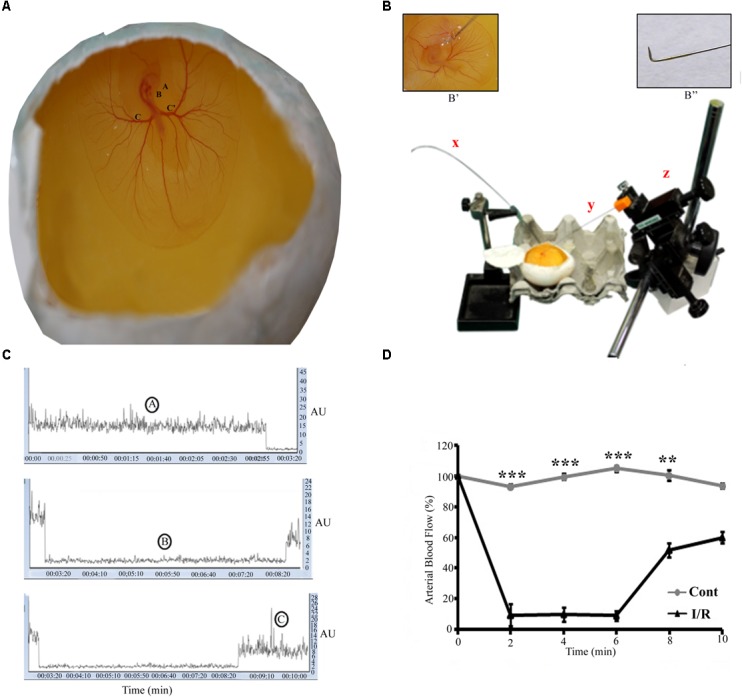
Representative pictures of the I/R setup for Hook Model. **(A)** A 72-h Leghorn chick embryo in an *in ovo* culture, Annotations: A = eye, B = heart, C = left vitelline artery, C’ = right vitelline artery. The rectangle area represents the site of the occlusion. **(B)** The typical setup to induce I/R, x = laser Doppler flow probe, y = spinal needle (insight picture shows the bent edge of the spinal needle), z = micromanipulator. The magnification **B’** shows the enlarge picture of the artery while occlusion. **B”** shows the spinal hook (arrow) inserted beneath RVA (arrow-head) to lift up the artery. **(C)** The changes in the flux during normoxia **(A)**, ischemia **(B)**, and reperfusion **(C)**. **(D)** The baseline arterial blood flow in I/R treated RVA vs. control RVA, and the changes observed in I/R RVA during ischemia-reperfusion (*n* = 12/group/experiments). Control represents Sham. ^∗∗^*P* < 0.01 and ^∗∗∗^*P* < 0.001.

Unborn embryos, such as chick, zebrafish, and *Xenopus*, have been extensively used in biomedical research. Chick embryos are widely used because of their ready accessibility, ethical acceptability, relatively large size, cost effectiveness, and fast growth (Seabra and Bhogal, 2010). Chick embryos have played a vital role in anatomical, embryological, developmental biology studies and they are an effective model for blood circulation research (Harvey, 1628; Lee et al., 2011; Smith et al., 2016). Furthermore, the blood vessel network of chick embryos can be a repository system for implanted human cells without any rejection (Wilson and Chambers, 2004; Deryugina and Quigley, 2009). Because the third day chick embryos possess a well-defined circulatory system, we selected a 72-h chick embryo as an appropriate model to study the I/R mechanism (**Figure 1A**). The model used (hereafter referred to as the Hook I/R model) in the present study can effectively mimic all downstream pathway, e.g., oxidative and inflammatory pathways. Our model is simple, reproducible, and can be used for drug screening, and for routine I/R studies.

## Materials and Methods

### Ethics Statement

The experimental protocol for the use of chick embryos was submitted to the Era’s Lucknow Medical College and Hospital’s Institutional Animal Ethical Committee, which issued a written waiver stating that according to the Committee for the Purpose of Control and Supervision of Experiments on Animal (CPCSEA) no formal approval was necessary to perform these experiments. Though, Standard Operating Procedures were followed to minimize any possible suffering by embryos.

### Materials and Equipment

We used fertilized White Leghorn chicken eggs, 37°C egg incubator (Lab Guard, India), Kim-wipe, 70% ethanol, surgical scissors, 26G needle and syringe, laser Doppler blood flow imaging system (Moors Instrument, United Kingdom), micromanipulator (Narishige, United States), and surgical microscope (Olympus, Japan). The primary antibodies used were rabbit polyclonal anti-HIF1α (NB100-449, Novus Biologicals), mouse polyclonal anti-LC3 (SC16756, Santa Cruz), rabbit polyclonal anti-Beclin1 (24352, SAB), rabbit polyclonal anti-SOD 1 (3458-100, Biovision), rabbit polyclonal anti-SOD 2 (NB100-1992SS, Novus Biologicals), rabbit monoclonal Caspase-1 (ab179515, Abcam), mouse monoclonal Caspae-3 (NB100-56708SS, Novus Biologicals), mouse monoclonal Cathepsin B (ab58802, Abcam), rat monoclonal LAMP1 (ab25245, Abcam), mouse monoclonal LAMP-2 (NBP2-22217SS, Novus Biologicals), rabbit monoclonal IFNγ (ab133566), rat monoclonal anti-NLRP3 (MAB7578-SP, Novus Biologicals), rabbit polyclonal anti-ASC (PA5-50915, Invitrogen), mouse monoclonal anti-IL-1β (701304, Invitrogen), rabbit polyclonal anti-Ambra 1 (GTX17003, Genetex), rabbit polyclonal anti-ATG7 (PA535203, Themofisher), rabbit polyclonal anti-SQSTM1 (PA520839, Invitrogen), rabbit polyclonal anti-ORP150 (NBP2-14113, Novus Biologicals), rabbit polyclonal anti-NF-Kβ (51-0500, Invitrogen), rabbit polyclonal anti-TNFα (NB600-587SS, Novus Biologicals), and rabbit polyclonal anti-GAPDH (ITI5052, GBIO). The secondary antibodies were HRP-conjugated donkey anti-rabbit (Jackson ImmunoResearch, 126333) and HRP-conjugated goat anti-mouse (Cell Signaling Technology, 70765) antibodies. An ECL chemiluminescence kit (Biorad, 170-5060), RIPA (Sigma Aldrich, R0278), protease inhibitor (Cell Signaling Technology, 5872), 2-thiobarbituric acid (Sigma Aldrich, T5500), sulfanilamide (Sigma Aldrich, S9251), and N-1-napthylenediamine (Sigma Aldrich, N9125) were obtained from the indicated sources.

### Chick Eggs and Embryos

Fertilized White Leghorn chicken were procured from Central Avian Research Institute, Bareilly, Uttar Pradesh, India, and where kept in our in campus poultry farm. Zero-day-old eggs were incubated in a 37°C egg incubator at 60–65% humidity for 72 h. After 24 h, the eggs were taken out for layering: 5–6 mL of albumin was drawn out. On the third day of incubation, the eggs were removed from the incubator, and windowing was performed to assess the embryo as described below.

### Inducing Ischemia in the Chick Embryo Through the Hook I/R Method

Ischemia was induced in the chick embryo at 72 h of development. The RVA, the artery responsible for carrying oxygen and nutrients to the embryo, was pulled out by a spinal needle [Ramsons, India; 25GA, 3.50 IN (90.51 × 90 mm)] under the guidance of a surgical microscope (**Figure 1** and **Supplementary Figure S1**). Two small holes were created on the right and left side of the RVA in the sac (**Supplementary Figure S2**), with the help of 18G needle. A hook was custom-designed at the base of the spinal needle (**Figure 1B**), attached to the micromanipulator (Narishige, Japan) to lift up the RVA. The length of the bent part was approximately 1 ± 0.5 mm. The needle was inserted beneath the RVA through the two small holes created, to lift up the artery. The artery was raised gently till the Doppler reading for arterial blood flow showed a decreased of 80–90% of the baseline value. The Doppler was positioned directly onto the RVA at a distance of 5 ± 1 mm post-ischemic site (**Supplementary Figure S2**). Post-ischemia the artery was released slowly and the spinal needle was retracted back with the help of the micromanipulator. During reperfusion, the Doppler reading reached a normal value (**Supplementary Figure S3**). After 5 min of ischemia, reperfusion was allowed for the next 5.5 h. After occlusion, a few drops of phosphate buffer saline were added to the yolk to prevent drying; subsequently, the egg was sealed with cello-tape and placed back into the 37°C incubator. After 5.5 h, the egg was taken out of the incubator for further treatment. For the control experiments, Sham without surgery was taken.

#### Vascular Blood Flow Imaging

To validate the model, the blood flow in the RVA was monitored before, during, and after ischemia by using a laser Doppler flow meter (moorVMS-LDF1, Moor Instruments, United Kingdom; **Figure 1C**).

### Biochemical Analysis

#### NO Estimation

NO production was evaluated by measuring the level of nitrite (an indicator of NO) in the supernatant of right vitelline artery of control and experimental ischemic chick embryos using Griess reagent (Green et al., 1982). Briefly, 150 μL of tissue supernatant was mixed with 150 μL of Griess reagent [0.1% *N*-(1-naphthyl) ethylene diamine dihydrochloride, 1% sulfanilamide, and 2.5% H_3_PO_4_]. After incubation at room temperature in the dark for 10 min, absorbance was measured on a microplate ELISA reader (Biorad, United States) at 540 nm.

#### Thiobarbituric Acid Reactive Substances Assay

LPO was estimated by measuring thiobarbituric acid reactive substances (TBARS, a marker for LPO) following the method of Utley et al. (1967), with some modification. In brief, 0.2 mL of supernatant was pipetted into a 2.0 mL flat bottom eppendorf tube and was incubated at 37°C in a metabolic water bath shaker at 120 strokes up and down; another 0.2 mL of the same supernatant was pipetted into a microcentrifuge tube and was incubated at 0°C. After 1 h of incubation, 0.4 mL of 5% TCA and 0.4 mL of 0.67% TBA were added to both samples (i.e., those incubated at 0 and 37°C). The reaction mixture was centrifuged at 3000 *g* for 15 min. The supernatant was transferred to another test tube and placed in a boiling water bath for 10 min. Thereafter, the test tubes were cooled, and absorbance was read at 535 nm. The LPO concentration is expressed as nmol of TBARS formed/h/mg protein using the molar extinction coefficient of 1.56 × 10^5^/M/cm.

### Western Blotting

The proteins (30 μg; loaded to each well) from the RVA and its adjoining tissue (**Supplementary Figure S2**), from the control and I/R groups were analyzed through western blotting. In brief, tissues were lyzed in ice-cold RIPA buffer (Sigma Aldrich, R0278) containing protease and phosphatase inhibitors (Cell Signaling Technology, 5871S) and were homogenized in tissue homogenizer in the same buffer used for cell lysis and centrifuged at 20,000 *g* for 20 min at 4°C. The protein concentration was measured using a Pierce BCA protein assay kit (Life Technologies, 23225). The proteins were then fractionated through SDS-PAGE and transferred on to a PVDF membrane (Biorad, 1610177). The membranes were blocked with 5% non-fat milk, probed with primary antibodies overnight at 4°C, and incubated with HRP-conjugated secondary antibodies at room temperature for 1 h. Immunoblot bands were quantified using densitometry on ImageJ. Densities were normalized to control treatment, and relative fold changes were normalized to GAPDH.

### DNA Gel Electrophoresis for DNA Damage

The I/R and control samples RVA (20 mg) were ground with liquid nitrogen, and DNA extraction buffer (pH 7.5) and proteinase K were added. The samples were then incubated in a heat block at 60°C for 30 min and centrifuged at 13,000 rpm for 15 min. Ammonium acetate (5M) and isopropanol were added to the supernatant, which was again centrifuged at the same speed (13,000 rpm) for 5 min. The DNA pellet was then washed with ethanol and re-suspended in 50 μL of TE buffer containing RNAase for the experiment. The isolated DNA (2 μg) from each treatment was examined for DNA damage by conducting DNA electrophoresis in 0.2% agarose gel in Tris/acetate buffer at 15 V for 2 h. At the end of electrophoresis, DNA was visualized in Chemidoc (Las500, GE).

### Drug Treatment

For drug treatment, the RVA was excised after 1 h of I/R, placed in Ringer’s solution (NaCl, KCl, CaCl_2_, pH = 7.4) containing anti-ischemic molecules [MD (Sigma Aldrich, M5199) and TMZ (i.e., 1-(2,3,4-trimethoxy benzyl); Sigma Aldrich, 653322)]; an anti-inflammatory molecule (MCC950), a ROS inhibitor (NAC; Sigma Aldrich, A7250), and their respective controls and incubated at 37°C for another 4.5 h.

### Statistical Analysis

**FIGURE 2:**
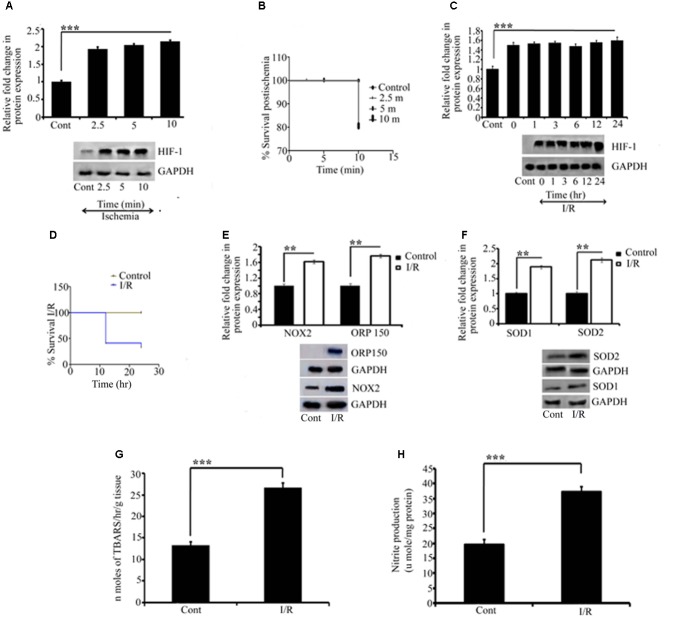
Representative pictures of Effect of I/R on ischemic and oxidative parameters. Effect of I/R and the activation of oxidative stress were measured by quantifying HIF1α, NOX2, ORP150, SOD1, and SOD2 expression. Initially to measure the effect of ischemia, HIF1α was quantified for different time point for which RVA was occluded **(A)**. All the occlusion time point showed significant differences compared to control (^∗∗∗^*P* < 0.001). **(B)** The Kaplan Meier survival curve for the embryos receiving ischemia at different time points compared to the control (^∗^*P* < 0.05; *n* = 12/group/experiment). The effect of reperfusion was further quantified by HIF1α. A significant change as compared to the control was observed in all the time point since reperfusion is allowed (^∗∗∗^*P* < 0.001). No significant differences were observed in-between the ischemia and reperfusion time points **(C)**. Kaplan Meier survival curve for the reperfusion for different time points compared to the control showed a significant difference between (^∗^*P* < 0.05; *n* = 12/group/experiment) **(D)**. **(E,F)** The changes in NOX2 (^∗∗^*P* < 0.01), ORP150 (^∗∗^*P* < 0.01), SOD1 (^∗∗^*P* < 0.01), and SOD2 (^∗∗^*P* < 0.01) compared to their respective controls. Results represent mean ± SE (*n* = 3). The graph shows the densitometry quantification of western blot bands. **(G)** The changes in the level of TBARS after 6 h of I/R. Right, I/R vessels showed significant changes as compared to the right control (^∗∗∗^*P* < 0.001). No changes were observed in left control vitelline arteries vs. right control. **(H)** The changes in NO production in right I/R vs. right control vessels (^∗∗∗^*P* < 0.001). Results represent mean ± SE (*n* = 3). Control represents Sham in all the experiments. ^∗^*P* < 0.05, ^∗∗^*P* < 0.01, and ^∗∗∗^*P* < 0.001.

**FIGURE 3:**
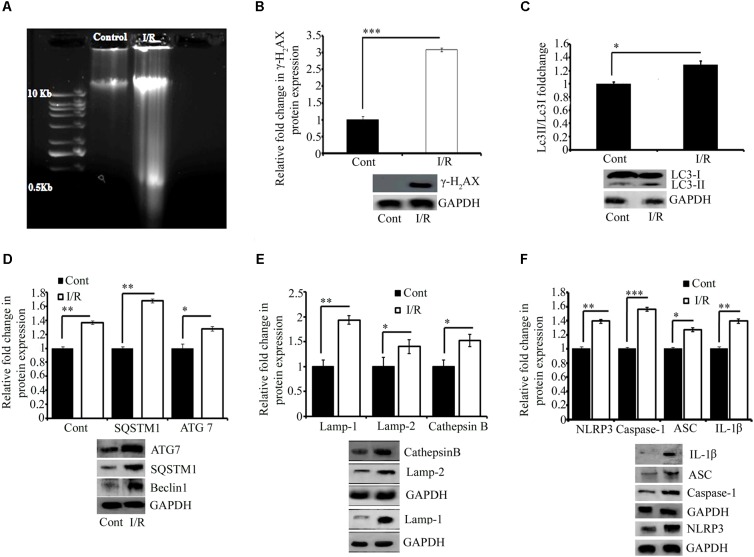
Representative pictures of effect of I/R on DNA damage, cell survival, and inflammation. **(A)** DNA gel electrophoresis diagram. **(B)** The phosphorylation of H2AX in response to I/R-induced DNA damage (^∗∗∗^*P* < 0.001 vs. control). Western blot analysis expression level of LC3 I/II (^∗^*P* < 0.05) **(C)**, Beclin1 (^∗∗∗^*P* < 0.001), SQSTM1 (^∗^*P* < 0.05), and ATG7 (^∗∗^*P* < 0.01) **(D)** and lysosomal associated proteins Lamp1 (^∗∗^*P* < 0.01), Lamp2 (^∗^*P* < 0.05), and Cathepsin B (^∗^*P* < 0.05) **(E)** in total protein extract from RVA of I/R treated vs. control group. **(F)** The expression of NLRP3 (^∗∗∗^*P* < 0.001), Caspase-1 (^∗∗^*P* < 0.01), ASC (^∗∗^*P* < 0.01), and IL-1β (^∗∗^*P* < 0.01). Results represent mean ± SE (*n* = 3). The graph shows the densitometry quantification of western blot bands. Here control group represented Sham group. ^∗^*P* < 0.05, ^∗∗^*P* < 0.01, and ^∗∗∗^*P* < 0.001.

**FIGURE 4:**
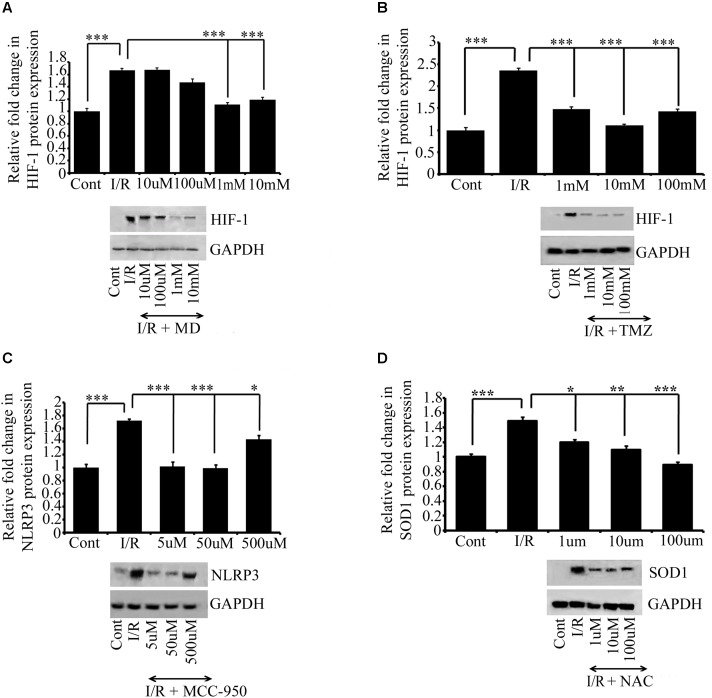
The effect of drug treatments on I/R RVA. Western blot analysis for MD **(A)**, TMZ **(B)**, MCC950 **(C)**, and NAC **(D)** shows that MD (1 mM, ^∗∗∗^*P* < 0.001) and TMZ (1 mM, ^∗∗∗^*P* < 0.001). Similarly, significant changes were found in the expression of MCC950 (5 μM, ^∗∗∗^*P* < 0.001) and NAC (100 μM, ^∗∗∗^*P* < 0.001) attenuates I/R mediated changes in I/R group compared to Ischemic group. Results represent mean ± SE (*n* = 3). The graph shows the densitometry quantification of western blot bands. Control group represents Sham group. ^∗^*P* < 0.05, ^∗∗^*P* < 0.01, and ^∗∗∗^*P* < 0.001.

The results were statistically evaluated with different tests. All the experimentations have been done three times as *n* = 3/group/experiments, unless otherwise indicated. Significant differences between the experimental groups were analyzed with student’s t test and with one-way ANOVA followed by Newman–Keuls multiple comparison test. Differences were considered statistically significant when *p* < 0.05. All data were presented as means ± SE. Student’s *t*-test was used to analyze data presented in **Figures 1D**, **2E–H**, **3B–F** and **Supplementary Figures S4**, **S5**. One-way ANOVA followed by Newman–Keuls multiple comparison test was used to analyze data in **Figures 1A,C**, **4**. Graph Pad Prism 5 software was used to calculate all the above data.

## Results

### Chick Embryo Hook I/R Model

Ischemia-reperfusion was induced in a chick embryo by occluding the right vitelline artery (RVA) for 5 min; after occlusion, the artery was released for the next 5.5 h. Functional changes in vascularization during the I/R period were mapped through laser Doppler perfusion imaging, which is widely used for microcirculatory imaging in human and rodents; however, to the best of our knowledge, this is the first instance in which this technique has been used to monitor blood flow in a chick model. The flux intensity of the control and I/R groups was measured, and a significant difference observed in the blood flow of the two groups (**Figure 1D**). During ischemia, we observed a flux intensity drop of >80% of the baseline normoxia level. The finding was consistent in all chick embryos employed; those with a lower drop (<80%) were discarded. The flux intensity reached a normal value after reperfusion was allowed (**Supplementary Figure S3**). Furthermore, to validate the chick embryo I/R Hook model, we employed western blotting and biochemical assays.

### Western Blotting Analysis for I/R Injury

Elevated expression of HIF1α is considered a protective measure mediated by the cell to protect itself against I/R-inflicted damage (Bergeron et al., 1999; Lee, 2000; Baranova et al., 2007). Here, we measured HIF1a expression, in the ischemic chick embryo model, as an early marker of ischemic tissue damage. We initially quantified HIF1α expression after 2.5, 5, and 10 min of ischemia to the RVA. The result suggested that ischemia induced in the tissue even for such a short duration was sufficient to stabilize HIF1α expression (^∗∗∗^*P* < 0.001; **Figure 2A**) in chick embryo. The observed fold changes during 2.5, 5, and 10 min of ischemia were 0.9-, 1.03-, and 1.14-fold, respectively. Although the fold changes during 10 min of ischemia were slightly higher (non-significant), the Kaplan–Meier survival curve showed 100% viability within 5 min of treatment (**Figure 2B**). Thus, we applied 5 min of ischemia in subsequent experiments. Because the restoration of blood supply often causes more damage to the tissue than the ischemic episode itself, we analyzed the effect of reperfusion at different time intervals (**Figure 2C**): compared with control, the level of HIF-1α expression increased rapidly after ischemia (^∗∗∗^*P* < 0.001); however, no significant differences were observed between 0 and 24 h of reperfusion. Notably, no changes were observed in the reperfusion time window, and the maximum survival was obtained at 6 h (**Figure 2D**). Therefore, we used 6 h of reperfusion in subsequent experiments.

### Involvement of Oxidative Stress

Ischemia-reperfusion activates several processes with detrimental effects on the tissue, including the generation of reactive oxygen species (ROS). Because NADPH oxidase 2 (NOX2) is a major source of O_2_ and H_2_O_2_, we investigated the contribution of NOX2 to oxidative stress in the chick embryo on the third day after I/R by measuring the changes in NOX2 expression. Compared with control embryos, we obtained strong evidence that NOX2 augments the tissue injury by 61% in I/R embryos (^∗∗^*P* < 0.01; **Figure 2E**). Moreover, we examined the expression of ORP-150, a novel stress protein that is activated in the pathophysiology of ischemic (Bando et al., 2004; Kitano et al., 2004) and oxidative (Goswami et al., 2003; Ozawa et al., 2005) diseases. Compared with the control group, ischemia, followed by reperfusion, upregulated ORP150 expression by approximately 76% in the I/R group (^∗∗^*P* < 0.01; **Figure 2E**), which corroborated the aforementioned finding.

An important hallmark of I/R injury is the detrimental and strong oxidative stress resulting from the intrinsic antioxidant body defense systems’ response to ROS. Among the antioxidant defense systems, superoxide dismutases (SOD) are key antioxidant enzymes that provide the first line of defense against ROS by catalyzing the conversion of O_2_ to H_2_O_2_. Thus, we examined the activity of cytoplasmic and mitochondrial SOD in ischemic vessels. Compared with the control group, I/R enhanced the activity of cytoplasmic SOD1 by 80% (^∗∗^*P* < 0.01) and mitochondrial SOD2 by 112% (^∗∗^*P* < 0.01) in the I/R group (**Figure 2F**). According to I/R research, the damage to the affected tissue results from the oxidative stress induced by free radicals, and the measurement of lipid peroxidation is one of the most commonly used assays for evaluating radical-induced damage (Svingen et al., 1979; Sevanian and Hochstein, 1985). We evaluated the level of lipid peroxides in the I/R and control groups. Our study confirmed the formation of thio-barbituric acid reactive species in ischemic RVA compared with non-ischemic arteries (^∗∗∗^*P* < 0.001 vs. control; **Figure 2G**). We also estimated the nitric oxide (NO) level in I/R and control arteries using the Griess reagent, and the resulting pattern was the same as demonstrated in lipid peroxidation (^∗∗∗^*P* < 0.001 vs. control; **Figure 2H**).

### Model for DNA Integrity

Reports have documented that I/R can cause DNA damage in cells and tissues (Ge et al., 2017; Kim, 2017; Hu et al., 2018). Thus, we examined the effect of 5 min of ischemia, followed by 5.5 h of reperfusion, on the mechanical integrity of DNA. Profound fragmentation of DNA was evident in the I/R group compared with the control group, indicating the loss of DNA integrity (**Figure 3A**). To confirm the results, we quantified the expression of H2AX, a highly sensitive marker of double-stranded DNA damage that localizes to the site of the DNA break. Western blot analysis of H2AX indicated that the I/R group showed two fold higher DNA damage than that in the control group (^∗∗∗^*P* < 0.001; **Figure 3B**).

### Model for Cell Death Mechanisms

Ischemia-reperfusion activates autophagy (Chen et al., 2017;Yang et al., 2017; Huang et al., 2018; Peng et al., 2018; Xie et al., 2018). Thus, we investigated the effect of I/R on the autophagy mechanism in the chick embryo. As shown in **Figures 4A,B**, the expression of LC3 (autophagosome marker; ^∗^*P* < 0.05), Beclin1 (a central regulator of autophagy in mammalian cells; ^∗∗∗^*P* < 0.001 vs. control), p62 (cargo protein; ^∗^*P* < 0.05 vs. control), and ATG7 (required for basal autophagy; ^∗∗^*P* < 0.01 vs. control) of the I/R group was significantly higher than that of the control group (**Figures 3C,D**). We next examined the expression of the following autolysosomal proteins: lysosomal-associated membrane proteins LAMP1 and LAMP2, and Cathepsin B. Their expression pattern showed a strong correlation with the expression of initiation and cargo proteins (**Figure 3E**). After 5 min of ischemia, followed by 5.5 h of reperfusion, the expression of LAMP1, LAMP2, and Cathepsin B increased by 94, 40, and 52% in I/R arteries, respectively. We also tried to observe the effect of I/R on apoptosis (**Supplementary Figure S4**) and the outcome indicate that the model may also be used to study the other pathways of cell death (e.g., apoptosis).

### Chick Embryo as a Model for I*/*R Inflammation

To verify the efficacy of this model for conducting inflammatory research, we evaluated the expression of the NOD-like receptor pyrin domain-containing protein 3 (NLRP3) inflammasome pathway, and pro-inflammatory cytokines NF-kβ and IFNγ, involved in exaggerating inflammation. This study provided evidence for the activation of both the NLRP3 inflammasome (**Figure 3F**) as well as NF-kβ and IFNγ (**Supplementary Figure S5**) in response to I/R induced in the RVA. Six hours of I/R increased the expression of NLRP3, cleaved caspase-1, ASC, and cleaved IL-1β by 1.4-, 0.9-, 0.83-, and 1.3-fold, respectively, and the changes in the levels of NF-kβ and IFNγ were 1.2-and 0.8-fold, respectively.

### Treatment With Meldonium Dihydrate, Trimetazidine, MCC950, and *N*-Acetyl Cysteine

The main advantage of generating a model is that it can be used to test the efficacy of drugs. To verify that the Hook I/R model can be used for drug screening, we evaluated the protective effect of meldonium dihydrate (MD), trimetazidine (TMZ), MCC950, and *N*-acetyl cysteine (NAC). Several doses of these drugs were tested; the doses were chosen arbitrarily (with 10-fold increases in the concentration of the primary dose), or the doses selected were intermediate between the dose selected for cell culture and that used in animal models in other studies. Six hours of I/R induced changes in the expression of HIF 1α and NLRP3 in the I/R group. However, compared with the I/R group, I/R induction was blocked in the drug-administered group treated with MD (1 mM, ^∗∗∗^*P* < 0.001; **Figure 4A**) and TMZ (1 mM, ^∗∗∗^*P* < 0.001; **Figure 4B**). Similarly, significant changes were found in the expression of MCC950 (5 μM, ^∗∗∗^*P* < 0.001; **Figure 4C**) and NAC (100 μM, ^∗∗∗^*P* < 0.001; **Figure 4D**) treated groups respectively, as compared to I/R group. The expression of the respective proteins in the I/R group and the groups treated with the MD, TMZ, MCC950, and NAC indicated that ischemia, inflammation and oxidative stress, respectively, were significantly ameliorated.

## Discussion

Ischemia-reperfusion research focuses on developing therapeutic strategies to prevent cell death and improve recovery. In this study, to overcome the existing challenges in I/R research, we developed an I/R chick embryo model. We aimed to develop a reliable and reproducible model of I/R to study stress signals (e.g., oxidative and inflammatory stress). In addition to the high output, simplicity, and flexibility of the model for routine analysis, the model can be used for short-term stem cell homing studies (ongoing studies).

Ischemia is the deprivation of blood supply; hence, tissues and the body parts are deprived of oxygen and nutrients (Hunt et al., 1985; Mansfield et al., 1997; Madarame et al., 2013; Matsubara et al., 2015), and ischemia is typically caused by the narrowing or blockage of blood vessels or arteries. Reperfusion is the only intervention that can reliably reduce the infarct size in animals and humans (Hale et al., 2013; Wei et al., 2016; Borges and Verdoorn, 2017; Misir et al., 2017; Sunagawa et al., 2018). Several preclinical cell culture and animal testing models are currently available to identify, assess, and prioritize the day-to-day new preventive and therapeutic inventions against I/R injury. However, each model has limitations; for example, cell culture models lack whole physiology, immune system, and microvasculature; similarly, rodents studies are highly regulated and supervised, costly, time-consuming, and have ethical issues. In the present study, we investigated, for the first time, the possibility of using chick embryos as a model organism to study I/R. Previous efforts have been made to develop a chick embryo-based flow-manipulation model for studies of shear stress (Hogers et al., 1999; Dittmar et al., 2006; Groenendijk et al., 2007; Wang et al., 2009). These studies have evaluated the consequences of low blood flow for embryo development and cardiovascular malformation. Recently, Majumder et al. (2010) used ligation to induce ischemia in a chick embryo; however, the model lacked the reperfusion phenomenon. Thus, in the present study, we developed a model that can efficiently recapitulate the I/R events. To validate the model, we employed laser Doppler perfusion imaging. The measuring capacity of Doppler imaging relies on the structure, density, and depth of the capillaries. In our study, the laser probe was directly adjusted onto the RVA, so that the flux intensity was measured with maximum accuracy. The Doppler reading showed a drop of ≥80% of the baseline during ischemia in the I/R group.

In chick embryos, vitelline vessels are responsible for the circulation of blood from embryos to the yolk sac. Through vitelline circulation, embryos obtain nutrients from the yolk and diffused oxygen from air; hence, blocking any of the vitelline vessels can interfere with nutrient and oxygen transport. Based on these facts, we induced ischemia by occluding the blood supply to the RVA for 5 min, followed by reperfusion for another 5.5 h. The present study clearly demonstrated that the occlusion of the RVA induced ischemia, as evident by laser Doppler imaging and HIF1α expression. Oxidative and inflammatory stresses are among the pathophysiological changes postulated to occur in response to I/R (Aragno et al., 2003; Chen et al., 2007; Wong and Crack, 2008; Granger and Kvietys, 2015; Kurian et al., 2016; Rovcanin et al., 2016; Gao et al., 2017). Thus, we examined the expression of the following proteins involved in oxidative stress and inflammation: NOX2 (Lou et al., 2018; Zhang et al., 2018), ORP150 (Kitano et al., 2004; Ye et al., 2013), SOD1 (Jiang et al., 2015; Dibas et al., 2018), SOD2 (Haines et al., 2010; Xu et al., 2010), NLRP3 (He et al., 2017; Liu et al., 2018), NF-κβ (Su et al., 2017; Ye et al., 2017, 2018), and IFNγ (Sun et al., 2016; Ferhat et al., 2018). The high expression of these proteins in the present I/R model implied that even a short period of ischemia, followed by reperfusion, played a major role in exaggerating tissue damage. Furthermore, the induction of NF-κβ and IFNγ expression in the I/R embryo indicates that the present model can effectively be used to explore numerous inflammatory pathways involved in I/R.

Cells undergo cell death through many pathways, such as necrosis, apoptosis, and autophagy. Both apoptosis and autophagy are types of programmed cell death (PCD). Most knowledge on the role and regulation of PCD has been primarily obtained through three model systems: the nematode *Caenorhabditis elegans*, the fruit fly *Drosophila melanogaster*, and mouse (Fuchs and Steller, 2011). In this context, we tested the mechanism of cell survival and death in chick embryos. The expression of several proteins associated with autophagy was examined. The result suggested that 5 min of ischemia, followed by 5.5 min of reperfusion, promoted autophagy in chick arterial cells. The result indicated that a short period of I/R was sufficient to activate autophagy. Furthermore, our result indicated that the present model can be used to study all events associated with autophagy, and may also be employed to study other PCD pathways (**Supplementary Figure S4**).

Although many notable drug development achievements have been made in the last two decades in ischemic research, a key challenge in drug candidate screening is the lack of a suitable screening model, which can translate preclinical drug candidates into clinical success (Andreadou et al., 2008; Bahjat et al., 2017). In this study, we critically evaluated the role anti-oxidative, anti-ischemic, and anti-inflammatory drugs after 6 h of I/R. The result indicated that treatment with the anti-ischemic drugs MD (Sisetskii et al., 1992; Hayashi et al., 2000) and TMZ (Zhou et al., 2012; Yao et al., 2015) reversed the I/R response in the arteries. MCC950, a potent and selective inhibitor of the NLRP3 inflammasome (Coll et al., 2015; Salla et al., 2018), also protected the RVA against I/R in our model, corroborating the earlier reports of animal I/R models (Ye et al., 2017; Ismael et al., 2018). Additionally, we employed NAC, a scavenger of ROS, which was found to reverse the outcome of 6 h of I/R. Taken together, the results imply that the developed model can be effectively used to classify several drug types and their targets in I/R research.

In addition to I/R research, the present model can be employed for a range of other pathological mechanisms associated with I/R and for screening numerous drug types. Because of its high reproducibility, cost-effectiveness, and simplicity, we anticipate that our model can be a valuable resource for basic science and translational research, and that it can be widely used in I/R research. A direct infarct measurement could also been a good indicator of I/R mediated injury, and to map up the effect of various therapeutic drugs. Thus, we tried to quantify the infarct area, however, due to the delicate structure of the 72 h developed chicks we have yet not been able to measure the infarct area. Thus, further investigation is warranted for the comprehensive analysis of methods and pathways associated to I/R that we could not examine in this study.

## Author Contributions

SR contributed to experiment planning, conception, design, acquisition of data, analysis and interpretation of data, and drafting and revising manuscript critically. EF, AS, MAK, and PG contributed to acquisition of data. SR, TB, MK, and EF contributed to analysis and interpretation of data. EF and TB contributed to data preparation. AR provided the MCC950, reviewed the manuscript, and contributed to critical inputs to the experimentations.

## Conflict of Interest Statement

The authors declare that the research was conducted in the absence of any commercial or financial relationships that could be construed as a potential conflict of interest.

## Supplementary Material

FIGURE S1 Represent the schematic picture of the third day chick embryo I/R experimentation. **(A)** The day 0 processing. At the day 0, eggs were sterilized with 70% ethanol, and put into the 37°C egg incubator with 60–65% humidity. At day 1, the eggs were taken out for layering **(B)**. **(C)** A normal white Leghorn egg just before I/R surgery. **(D)** A typical setup to induce I/R in the chick embryo. The probe of the Doppler flow meter was put onto the ischemic RVA (5 ± 1 mm from the site of ischemia at distal end). **(E)** The magnified image of the setup: egg with micromanipulator and laser Doppler probe. **(F)** The insertion of the spinal need hook into the artery, and the lifting of the artery.

FIGURE S2 A typical picture of the third day embryo; the rectangle shows the site of excision of the tissue for western blotting, The star represents the site of the occlusion, and the holes created on the right and left side of the RVA are represented by two circles on the right and left sides of the artery, and is to insert the needle beneath the artery to lift it up. **(S2’)** The magnified image of **S2**. **(S2”)** The area of excision of tissue in the vicinity of RVA. And the straight line represents the RVA emerging from the embryo trunk. The star on the line denotes the position of laser Doppler flow probe. The intersection represents the site of occlusion. From the site of occlusion the arteries were excised up to 15 ± 1 mm (distal from the trunk), 5 ± 1 mm each on the left and right side of the artery, and 2 ± 1 mm toward the trunk. **S2”** is a representative of the right side of the chick embryo.

FIGURE S3 A typical laser Doppler flow meter signal measured on 3-days-old embryo chick artery from a control group chick **(A)**. **(B)** Events of baseline (1), immediate pre-lifting of RVA (2), during normoxia (3), immediate post-lifting of RVA (4), during ischemia (5), immediate pre-releasing of RVA (6), immediate post-reperfusion (7), during reperfusion (8) in ischemia treated group as recorded by laser Doppler flow meter.

FIGURE S4 Western blot analysis expression level of Caspase-3 (^∗∗∗^*P* < 0.01) in total protein extract from RVA of I/R treated vs. control group. Results represent mean ± SE (*n* = 3). The graph shows the densitometry quantification of western blot bands.

FIGURE S5 Western blot analysis expression level of NF-kβ (^∗∗∗^*P* < 0.001) and IFNγ (^∗∗∗^*P* < 0.001) (**Figure 4C**) in total protein extract from RVA of I/R treated vs. control group. Results represent mean ± SE (*n* = 3). The graph shows the densitometry quantification of western blot bands.
