# Supplementary figures and images for: Chick Embryo: A Preclinical Model for Understanding Ischemia-Reperfusion Mechanism
Source: Front Pharmacol. 2018 Sep 21;9:1034. doi: 10.3389/fphar.2018.01034 (PMC6160536; doi:10.3389/fphar.2018.01034)

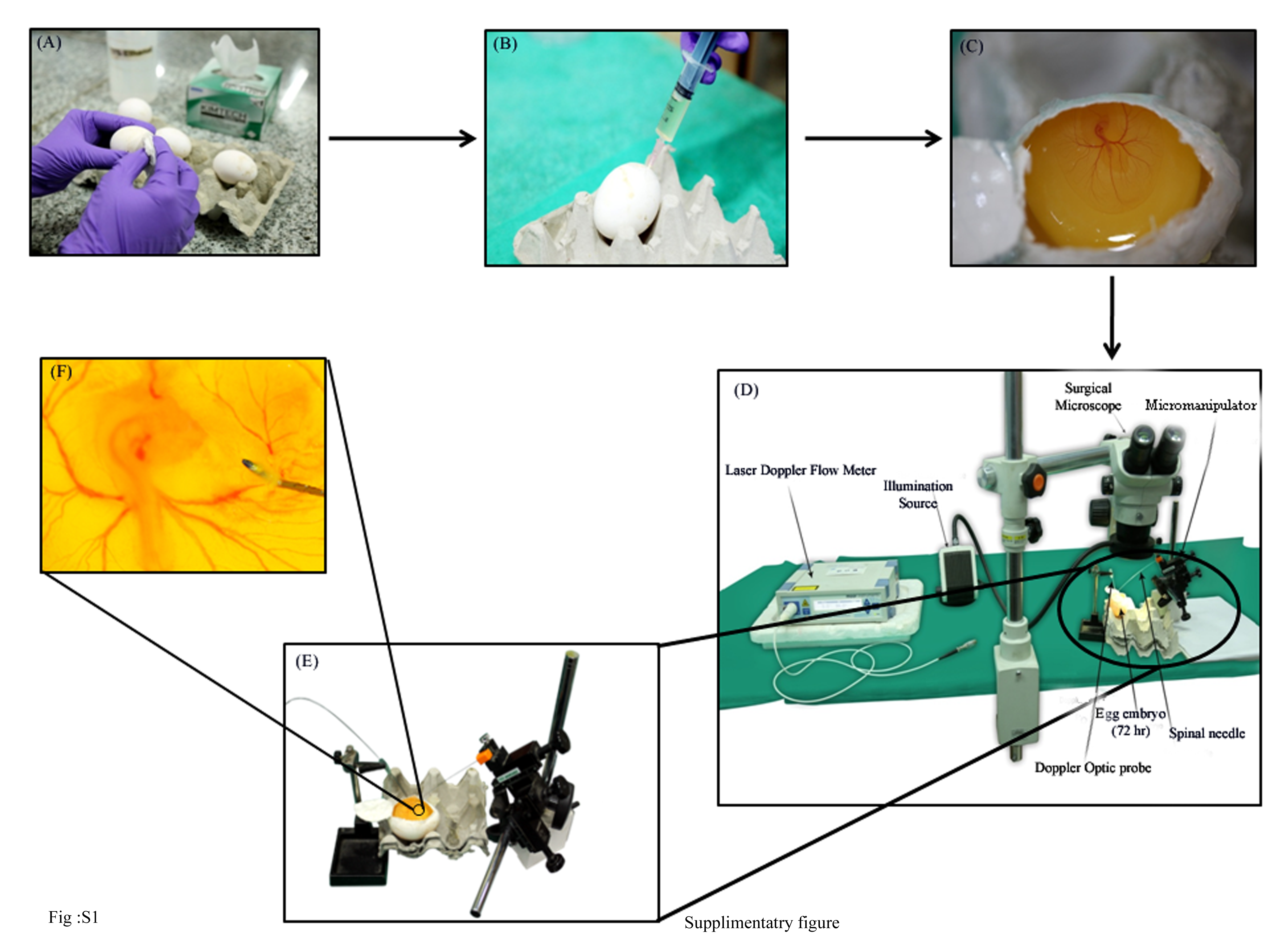

Supplement: FIGURE S1 — Represent the schematic picture of the third day chick embryo I/R experimentation. (A) The day 0 processing. At the day 0, eggs were sterilized with 70% ethanol, and put into the 37°C egg incubator with 60–65% humidity. At day 1, the eggs were taken out for layering (B). (C) A normal white Leghorn egg just before I/R surgery. (D) A typical setup to induce I/R in the chick embryo. The probe of the Doppler flow meter was put onto the ischemic RVA (5 ± 1 mm from the site of ischemia at distal end). (E) The magnified image of the setup: egg with micromanipulator and laser Doppler probe. (F) The insertion of the spinal need hook into the artery, and the lifting of the artery. [file Image_1.TIF]

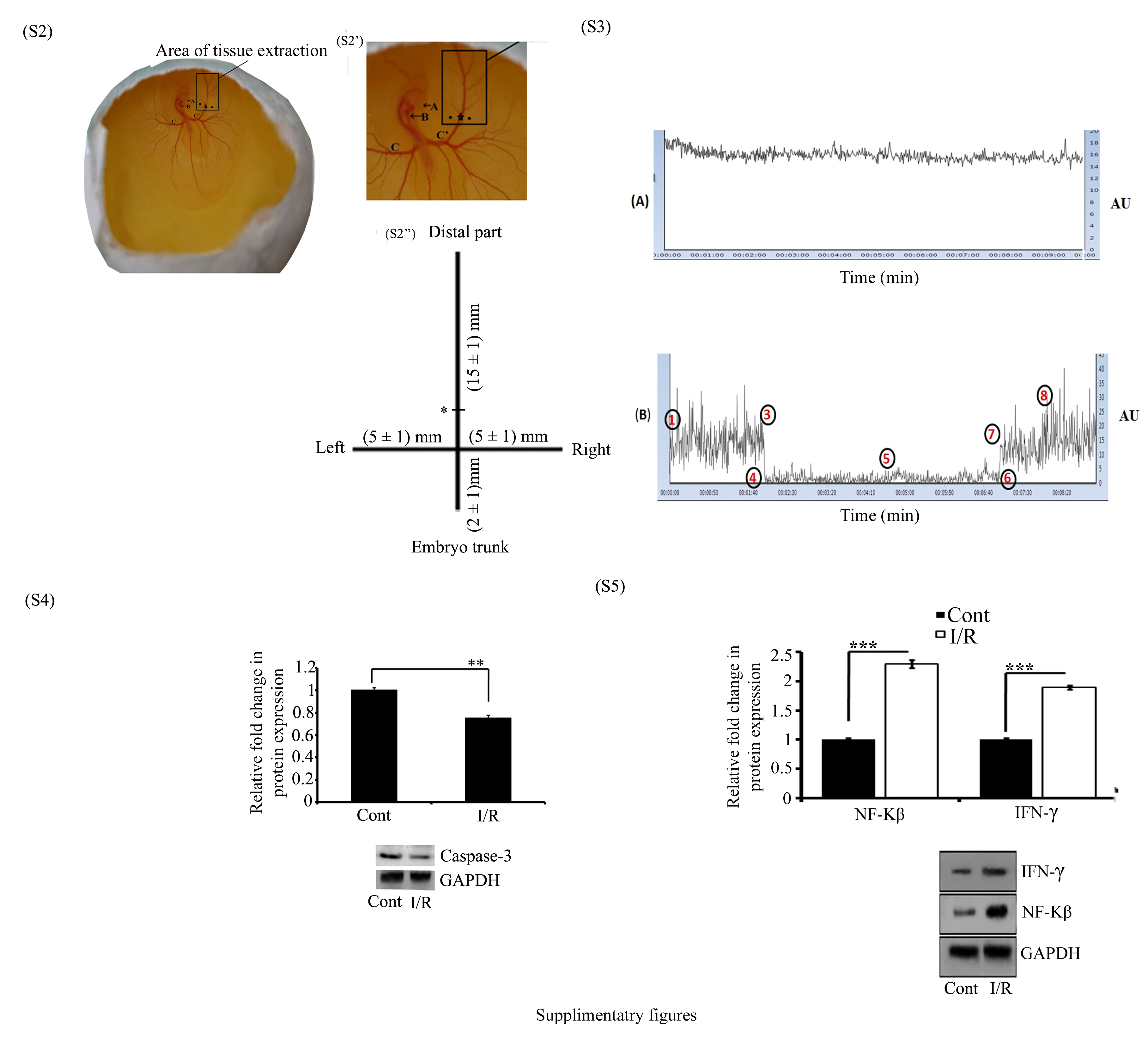

Supplement: FIGURE S2 — A typical picture of the third day embryo; the rectangle shows the site of excision of the tissue for western blotting, The star represents the site of the occlusion, and the holes created on the right and left side of the RVA are represented by two circles on the right and left sides of the artery, and is to insert the needle beneath the artery to lift it up. (S2’) The magnified image of S2. (S2”) The area of excision of tissue in the vicinity of RVA. And the straight line represents the RVA emerging from the embryo trunk. The star on the line denotes the position of laser Doppler flow probe. The intersection represents the site of occlusion. From the site of occlusion the arteries were excised up to 15 ± 1 mm (distal from the trunk), 5 ± 1 mm each on the left and right side of the artery, and 2 ± 1 mm toward the trunk. S2” is a representative of the right side of the chick embryo. [file Image_2.TIF]
